# Combination of metformin with other antioxidants may increase its renoprotective efficacy

**DOI:** 10.12861/jrip.2013.13

**Published:** 2013-06-01

**Authors:** Mahmoud Rafieian-Kopaei, Azar Baradaran

**Affiliations:** ^1^Medical Plants Research Center, Shahrekord University of Medical Sciences, Shahrekord, Iran; ^2^Department of Clinical Pathology, Isfahan University of Medical Sciences, Isfahan, Iran

**Keywords:** Metformin, Renoprotection, Diabetic nephropathy

**Implication for health policy/practice/research/medical education:**

Potential kidney protective effects of metformin, might have synergistic effect with some other antioxidants such as medicinal plants.

Recently, much attention has been made toward the possible renoprotective efficacy of metformin. Morales *et al*. observed that gentamicin-induced renal tubular injury is ameliorated by metformin (1). Reactive oxygen species play an important role in the nephrotoxicity of gentamicin, resulting in acute kidney injury (1) and gentamicin is a mitochondrial toxin that can imply its toxic effects when excreted by the kidney (1,2). To find the potential efficacy of metformin to protect the kidneys from gentamicin-induced acute kidney injury and also to examine whether delay treatment with metformin in acute kidney injury, exerts similar benefits on gentamicin renal toxicity in rats, we conducted a study on Wistar rats (3). We found that metformin was able to prevent and attenuate gentamicin-induced acute kidney injury. Hence, it might be beneficial in patients under treatment with this drug (3). More recently, to test the efficacy of co-administration of garlic extract and metformin for prevention of gentamicin-renal toxicity in Wistar rats, we conducted another study on 70 male Wistar rats (4). The result of this study indicates that metformin and garlic or their combination has both curative and protective effects against gentamicin nephrotoxicity. Hence, garlic extract could safely be used together with metformin to increase the antioxidant potency to ameliorate gentamicin-tubular toxicity (1-5). Likewise, Bruckbauer and colleagues conducted a study to evaluate the synergistic effects of metformin, resveratrol and hydroxymethylbutyrate on insulin sensitivity (6). They suggested that resveratrol-hydroxymethylbutyrate combined with metformin might act synergistically on AMP-activated protein kinase-dependent pathways, leading to increased insulin sensitivity, which might reduce the therapeutic doses of metformin necessary in the treatment of diabetes. However, this combination might also increase the antioxidant efficacy of metformin.

Nephropathy is one of the most important complications of diabetes mellitus and metformin has been widely used for the treatment of type 2 diabetes (7).

A study conducted by Kim *et al*., revealed that metformin was able to protect podocytes in diabetic nephropathy (7), while in diabetic nephropathy, there are various aspects of tubular cell injury due to glycosuria which needs further protection (7). Thus according to our results on protecting effects of metformin against tubular injury by restoring the biochemical alterations and modulation of oxidative stress on the tubules (3,4), we might conclude, other than potential protective effects of metformin on kidneys, it might have synergistic effect with some other antioxidants to exert its renoprotection (3,4). Indeed metformin consumption significantly attenuates the increased malondialdehyde and total reactive oxygen species generation and restores both enzymatic and non-enzymatic antioxidants (1,2). These findings potentiate the clinical use of metformin in the prevention of diabetic nephropathy. Thus, our data lend further evidence for the attribution of metformin in its renoprotective property, in addition to its well-known hypoglycemic action. In this regard, to understand the metformin renoprotective properties better, more experimental rat models or clinical studies are suggested.

## Authors’ contributions

MRK edited the draft. AB prepared the final manuscript.

## Conflict of interests

The authors declared no competing interests.

## Ethical considerations

Ethical issues (including plagiarism, misconduct, data fabrication, falsification, double publication or submission, redundancy) have been completely observed by the authors.

## Funding/Support

None.
